# Contribution of Soil Fauna to Foliar Litter-Mass Loss in Winter in an Ecotone between Dry Valley and Montane Forest in the Upper Reaches of the Minjiang River

**DOI:** 10.1371/journal.pone.0124605

**Published:** 2015-04-22

**Authors:** Yan Peng, Wanqin Yang, Jun Li, Bin Wang, Chuan Zhang, Kai Yue, Fuzhong Wu

**Affiliations:** Long-term Research Station of Alpine Forest Ecosystem, Key Laboratory of Ecological Forestry Engineering, Institute of Ecology & Forestry, Sichuan Agricultural University, Chengdu 611130, Sichuan, PR China; Helmholtz Centre for Environmental Research (UFZ), GERMANY

## Abstract

Litter decomposition during winter can provide essential nutrients for plant growth in the subsequent growing season, which plays important role in preventing the expansion of dry areas and maintaining the stability of ecotone ecosystems. However, limited information is currently available on the contributions of soil fauna to litter decomposition during winter in such ecosystems. Therefore, a field experiment that included litterbags with two different mesh sizes (0.04 mm and 3 mm) was conducted to investigate the contribution of soil fauna to the loss of foliar litter mass in winter from November 2013 to April 2014 along the upper reaches of the Minjiang River. Two litter types of the dominant species were selected in each ecosystem: cypress (*Cupressus chengiana*) and oak (*Quercus baronii*) in ecotone; cypress (*Cupressus chengiana*) and clovershrub (*Campylotropis macrocarpa*) in dry valley; and fir (*Abies faxoniana*) and birch (*Betula albosinensis*) in montane forest. Over one winter incubation, foliar litter lost 6.0%-16.1%, 11.4%-26.0%, and 6.4%-8.5% of initial mass in the ecotone, dry valley and montane forest, respectively. Soil fauna showed obvious contributions to the loss of foliar litter mass in all of the ecosystems. The highest contribution (48.5%-56.8%) was observed in the ecotone, and the lowest contribution (0.4%-25.8%) was observed in the montane forest. Compared with other winter periods, thawing period exhibited higher soil fauna contributions to litter mass loss in ecotone and dry valley, but both thawing period and freezing period displayed higher soil fauna contributions in montane forest. Statistical analysis demonstrated that the contribution of soil fauna was significantly correlated with temperature and soil moisture during the winter-long incubation. These results suggest that temperature might be the primary control factor in foliar litter decomposition, but more active soil fauna in the ecotone could contribute more in litter decomposition and its related ecological processes in this region.

## Introduction

Global warming and human disturbance have increased drought conditions worldwide, and the area of arid and semi-arid land is expanding at an overwhelming rate [1]. As a functional transition region and buffer zone of environmental change, the ecotone between a dry valley and montane forest provides important ecological functions, such as conservation of water and nutrients and modulation of micro-climate, but also hinders the upward advance of arid regions [2]. Since the majority of aboveground net primary production of a terrestrial ecosystem is returned to the soil as plant litter [3,4], the decomposition of plant litter is one of the most important sources of carbon (C) and nutrients for primary productivity in terrestrial ecosystems [5]. Generally, the physical and biochemical environment, litter quality, and soil fauna communities and activities are the primary factors that modulate litter decomposition [6,7]. Previous studies have suggested that soil fauna exhibit certain activities in winter and provide obvious or significant contributions to litter decomposition [8]. Moreover, it has been well documented that the biodiversity and activity of soil fauna in ecotone were much higher compared with those adjacent ecosystems [9,10]. As a result, soil fauna in ecotone may contribute more to litter decomposition and other related ecological processes. However, although several studies have investigated litter decomposition in ecotones [11,12], few attentions have been paid on the contribution of soil fauna to litter decomposition in ecotone, especially such ecosystems between montane forest and dry valley.

Based on our investigations and previous studies [13–15], we designated the entire winter into three stages. The stage from litterfall to time when soil completely freezes is “pre-freezing period” which is characterized by frequent freezing and thawing event as temperatures fall to freezing point. The subsequent stage is designated “freezing period” when temperatures remain below 0°C. The third stage is the “thawing period” at which time soil thawing takes place with an increase in temperature in early spring. Compared with the continuous dry conditions in the dry valley and earlier freezing in the montane forest during pre-freezing period before the formation of snow cover, the higher moisture content in winter in the ecotone ecosystem can maintain a richer soil fauna diversity and a larger community [16]. With a decrease in temperature and increased snowfall, a thicker snow cover could provide a relatively stable environment for the activity of soil fauna in the ecotone and montane forest compared with the environment provided by the lower snowfall in the dry valley during the period when the soil is completely frozen [17]. As litter decomposition proceeds, the increase in temperature and precipitation in early spring can promote the activity of soil fauna in the ecotone and montane forest [18], although leaching during snow melting might inhibit the contribution of soil fauna to litter decomposition in the ecotone and montane forest ecosystems. However, as leaching usually plays a dominant role during the very early period since litter begins to decompose, the inhibiting effect of which on the contribution of soil fauna to litter decomposition in early spring may be small. On the other hand, soil fauna are mobile, have a strong ability to migrate, and can vary their food preferences, and they are highly sensitive to environmental changes (e.g., [8,19,20]), leading to ambiguity in terms of the function of soil fauna during litter decomposition and related ecological processes in the ecotone.

Along the upper reaches of the Minjiang River, the ecotone between dry valley and montane forest is located in the Mediterranean-Himalayan seismic zone. This fragile landscape ecosystem is formed by a dry valley, which is characterized by a harsh environment (e.g., long-term drought, strong wind), towards the lower edge and a montane forest, which is characterized by frequent mountain disasters (e.g., earthquake, landslide), infertile soil, a poor storage capacity for water and fertilizer, and intense interference by human activities, towards the upper edge [21]. The ecotone and montane forest has obvious characteristics of drought and freeze-thaw in winter. Differences in freezing and thawing dynamics during differing periods of winter can not only directly affect litter decomposition rate [22] and C release [13], but also may influence the activities of soil fauna during the process of litter decomposition. Moreover, soil fauna in different ecosystem may show differing rates of contribution to litter decomposition. As stated above that the environment may be more suitable for richer soil fauna diversity and community in ecotone during winter compared with dry valley and montane forest (e.g., higher temperature during freezing period than dry valley, and higher increase in temperature in thawing period than montane forest), we thus hypothesized that (*i*) the contribution rate of soil fauna to litter decomposition in the ecotone is higher than in the montane forest or dry valley, and (*ii*) the contribution rate varies among different periods of winter regardless of ecosystem types.

To test this hypothesis, a field experiment that included litterbags of two different mesh sizes (3 mm and 0.04 mm) was performed along the upper reaches of the Minjiang River. The contribution of soil fauna to foliar litter-mass loss was evaluated at multiple times throughout the winter season as decomposition proceeded and temperatures fluctuated. A single litter type or consistent litter type(s) used in different ecosystems was always useful to evaluate the effect of climate on litter decomposition (e.g., [6,23]), but such methods failed to predict the actual decomposition features of the litter type which is only specific to the site or ecosystem. Therefore, two litter types of the dominant species for each ecosystem were used in this study, for the goal to investigate the actual activities of soil fauna during litter decomposition in each ecosystem and their contributions to litter-mass loss. Specifically, in the ecotone, the dominant species were cypress (*Cupressus chengiana*) and oak (*Quercus baronii*); in the dry valley, the dominant species were cypress (*Cupressus chengiana*) and clovershrub (*Campylotropis macrocarpa*); and in the montane forest, the dominant species were fir (*Abies faxoniana*) and birch (*Betula albosinensis*). The objectives of this study were to examine the contribution of soil fauna to litter decomposition in the ecotone and compare this contribution to that of the adjacent dry valley and montane forest. The results could be useful in explaining details of litter decomposition in the ecotone, and provide efficient data on the maintenance of ecotone ecosystem structures and functions that prevent the expansion of arid land area.

## Materials and Methods

### Study area

Three sample sites were selected in Lixian County: montane forest (31.14° N, 102.53°E, *c*. 3000 m a.s.l.), ecotone (31.32° N, 103.26° E, *c*. 2140 m a.s.l.), and dry valley (31.31° N, 103.15° E, *c*. 1450 m a.s.l.). The studied montane forest is a representative subalpine forest, and the mean annual air temperature is approximately 3.6°C, with maximum and minimum air temperatures of 24.8°C and -15.8°C, respectively. The mean annual precipitation is approximately 850 mm. The freeze-thaw season begins in November and lasts until April of the following year [14,15]. The dominant tree species in this forest are fir and birch, which are interspersed with dense shrubs, and the soil is atteration. In the studied ecotone, the mean annual air temperature is 5.9–11°C, and the annual precipitation ranges from 500–900 mm. A seasonally alternating dry and wet climate occurs in this zone. The winter is dry and lasts from November to March of the following year, and it is characterized by strong sunshine, dry air, and a high water deficit [24]. The soil is alfisol, and the vegetation consists of xeromorphic or mesophytic shrubs and herbs. The dominant tree species in this ecotone are cypress and oak. In the studied dry valley, the mean annual air temperature and precipitation are approximately 11°C and less than 400 mm, respectively. The evaporation at the local climate station is greater than 800 mm [25]. Because of the harsh climate conditions and influence of the Foehn effect, the alternations between wetting and drying produce obvious effects in this zone. Low temperatures, dry air and low rainfall are the main characteristics of the dry valley. The soil here is barren and lacks organic matter [26]. The composition and structure of the vegetation is simple, and the plants are characterized by drought-enduring traits such as clumped growth, deep roots, small leaves, and thorns [27,28]. The plant species are mainly shrubs (e.g., clovershrub and *Sophora davidii*) and herbs (*Achnatherum heteropogon*). Historically, cypress has been the dominant species [29].

### Experimental design

The contribution of soil fauna to litter decomposition was determined by using the litterbag method with two different mesh sizes. In October 2013, freshly fallen leaves from fir and birch trees were collected from the forest floor of the sampling plots in the montane forest, and fresh foliar litter from cypress, oak, and clovershrub were collected from the sampling plots in the ecotone and dry valley. The collected fresh litter was air-dried for at least two weeks at room temperature to prevent structural damage to the litter during subsequent oven-drying. Subsamples of the foliar litter from each species were oven-dried at 65°C for 72 hours to calculate a moisture correction factor and then analyzed to determine the initial quality.

For each litterbag of each litter type, a mass of air-dried litter equivalent to 10 g of oven-dried sample was weighed and then placed in two kinds of 20 × 20 cm nylon bags, small mesh size (0.04 mm on both sides) and large mesh size (0.04 mm on the soil side and 3.00 mm on the reverse side), before the bag was sealed. The small mesh size bags could exclude almost all soil fauna except for very small members of the microfauna [20]. Usually, a mesh size chosen for access of the entire soil fauna is 5 mm in studies on the effect of soil fauna on litter decomposition [20,23]. According to our previous experiments, however, it is difficult to maintain the complete samples placed in the litterbags using such a coarse mesh size because of the small leaves of selected litter types (fir, cypress, and clovershrub) and the inconvenient traffic conditions in alpine regions, which would increase errors in calculating litter mass loss. Moreover, as a 2 mm mesh size litterbags can include meso- and macro-fauna according to Swift [20], and few soil fauna bigger than 3 mm were observed according to our previous investigation [30], we thus use a mesh size of 3 mm litterbags in the present study.

We explored 3 plots (5 × 5 m) from each of the selected sample sites to place the litter bags, and the distance between each individual plot was approximately 100 m. In total, 18 litterbags (two mesh types × three replicates × three sampling dates) for each species (except for cypress, which had 36 litterbags, with 18 for the ecotone and 18 for the dry valley plots) were placed on the forest floor of the 3 selected sampling plots on 12 November 2013. The air temperature and temperature in the litterbags were measured every two hours between 12 November 2013 and 22 April 2014 using DS1923-F5 iButton loggers (Maxim Integrated Products, Inc., Sunnyvale, CA, USA). To determine the temperature dynamics in the plots, the mean temperature, positive accumulated temperature, and negative accumulated temperature were calculated for each period [31,32]. The soil moisture was measured in each sampling plot as well. Soil moisture content was calculated as: soil moisture (%) = (wet weight—dry weight)/wet weight × 100. The litterbags were randomly retrieved from each plot on 22 December 2013 (pre-freezing period), 9 March 2014 (freezing period), and 22 April 2014 (thawing period). The selection of sampling dates was based on the changes in the freeze-thaw dynamics determined from previous field observations [14,30]. The retrieved litter was then oven-dried at 65°C for 72 h to determine the dry mass and calculate the litter-mass loss.

### Analysis and calculations

To determine the initial chemical characteristics of the litter, the oven-dried foliar litter was ground through a 1-mm sieve, and then C, nitrogen (N), phosphorus (P), cellulose and lignin analyses were performed according to Lu [33]. In brief, the C content was determined using the dichromate oxidation-ferrous sulfate titration method; the N content was determined using the Kjeldahl method; and the P content was determined using the molybdenum-blue colorimetric method.

Lignin and cellulose were measured using the acid-detergent lignin method [34]. For the foliar litter within each type of litterbag, the litter-mass loss rate (*L*_*t*_), mass loss rate driven by soil fauna for each period (*C*_*fau*_), contribution of soil fauna to the litter-mass loss for each period (*P*_*fau*_) and for the entire winter (*P*_*total*_) were calculated as follows [35,36]:

$$L_{t}\left(\%\right)=(M_{0}-M_{t})/M_{0}\times 100$$

$$C_{fau}\;\left(\%\right)=\left(L_{lt}-L_{st}\right)-\left(L_{l\left(t-1\right)}-L_{s\left(t-1\right)}\right)$$

$$P_{fau}\left(\%\right)=C_{fau}/(L_{lt}-L_{s\left(t-1\right)})\times 100$$

$$P_{total}\left(\%\right)=(L_{l}-L_{s})/L_{l}\times 100$$

where, *M*_*0*_ is the initial oven-dried litter mass (g); *M*_*t*_ is the dry mass of the litter remaining in the bag at each sampling period; (*L*_*lt*_ - *L*_*st*_) is the difference in mass loss rate between the two litterbag mesh sizes for sampling date “t”, and (*L*_*l(t-1)*_ - *L*_*s(t-1)*_) is the difference in the mass loss rate of the two litterbag mesh sizes for the sampling date “(t-1)” (t = 1, 2, 3); *L*_*lt*_ and *L*_*s(t-1)*_ are the litter-mass loss rates for the large mesh size litterbags of the sampling dates “t” and “(t-1)” (t = 1, 2, 3), respectively; and *L*_*l*_ and *L*_*s*_ are the litter-mass loss rates for the large and small mesh size litterbags of the last sampling date.

Prior to the statistical analysis, the data were tested for homogeneity of variance using Levene’s test and transformed when applicable [37]. An independent-sample t-test was performed to compare the litter-mass loss rate for a specific litter type between large and small mesh size litterbags. A one-way analysis of variance (ANOVA) was performed to test for differences in initial litter properties, litter-mass loss rate among periods, and contribution of soil fauna to litter-mass loss. A Pearson′s correlation analysis was used to assess relationships among factors at *P* < 0.05. All statistical analyses were performed using the SPSS 20.0 software package for Windows (SPSS Inc., Chicago, IL, USA).

### Ethics statement

Since March 2006, the Institute of Ecology & Forestry has had a permit from the Western Sichuan Forestry Bureau to conduct scientific experiments in Lixian County. The present study had negligible effects on the ecosystem functioning as the senescent fresh litter collected was only sampled at a very limited scale. The research did not involve measurements on humans or animals, and no endangered or protected plant species was involved.

## Results

### Initial litter quality

No significant difference was observed among all the litter types for the concentration of total C (Table 1). As to the concentration of total nitrogen (N) and total phosphorus (P), lignin concentration, and lignin/N ratio, although statistically dramatic differences were found for some species, there was at least one litter type from each site that showed insignificant difference (Table 1).

**Table 1:** Initial litter quality of the investigated litter types (mean ± SE, n = 3).

Species	TC (g/kg)	TN (g/kg)	TP (g/kg)	C/N	C/P	N/P	Cellulose (%)	Lignin (%)	Lignin/ Cellulose	Lignin/N
Fir	379.91 ± 7.97^a^	22.78 ± 0.57^a^	1.95 ± 0.37^a^	16.68 ± 0.32^b^	210.14 ± 41.45^a^	12.57 ± 2.45^a^	12.46 ± 0.59^b^	22.10 ± 3.57^bc^	1.79 ± 0.31^bc^	0.97 ± 0.16^b^
Birch	365.16 ± 5.18^a^	31.19 ± 1.38^bc^	1.02 ± 0.14^b^	11.74 ± 0.39^ac^	374.39 ± 60.01^b^	31.90 ± 5.07^b^	13.57 ± 0.70^b^	40.22 ± 3.37^a^	3.01 ±0.42^c^	1.30 ± 0.17^ab^
Cypress	357.57 ± 10.57^a^	26.55± 1.14^ac^	1.61± 0.01^ab^	13.49± 0.36^a^	222.09± 5.55^ab^	16.49± 0.62^a^	20.22± 0.40^c^	16.74± 0.82^b^	0.83± 0.05^b^	0.64± 0.06^a^
Oak	352.77 ± 21.43^a^	34.95± 1.87^bd^	1.78± 0.04^ab^	10.22± 1.15^c^	198.07± 7.22^a^	19.73± 1.55^ab^	7.53± 0.70^a^	32.92± 0.30^ac^	4.45± 0.41^a^	0.95± 0.05^ab^
Clovershrub	365.74 ± 25.82^a^	39.22 ± 1.09^d^	1.33 ± 0.04^ab^	9.31 ± 0.45^c^	276.75 ± 25.15^ab^	29.61 ± 1.30^b^	12.93 ± 0.12^b^	18.89 ± 1.70^b^	1.46 ± 0.13^b^	0.48 ± 0.05^a^

TC: total carbon; TN: total nitrogen; TP: total phosphorus.

Different lowercase letters indicate significant differences among different litter types (p < 0.05).

### Litter mass loss rate

**Fig 1:**
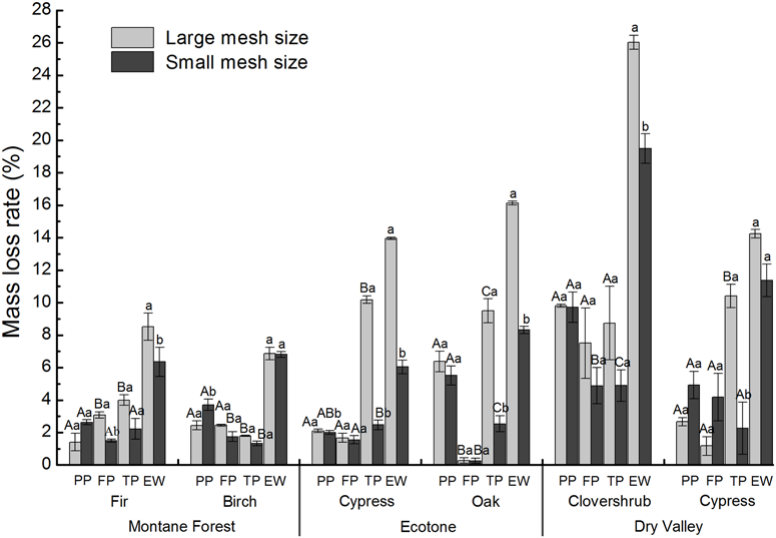
Foliar litter-mass loss rates from litterbags during winter for several litter types (mean ± SE, n = 3). Different capital letters indicate significant differences (*P* < 0.05) among the different periods for the same mesh size. Different lowercase letters indicate significant differences (*P* < 0.05) between different mesh sizes within a specific decomposition period. Abbreviations: PP = pre-freezing period; FP = freezing period; TP = thawing period; EW = the entire winter.

Over the entire winter incubation period, the foliar litter mass of the ecotone, dry valley, and montane forest decreased by 6.0%-16.1%, 11.4%-26.0%, and 6.4%-8.5% of their initial dry mass, respectively (Fig 1). The litter mass loss rates from the large mesh size litterbags were the highest during the thawing period of the winter regardless of litter type except for clovershrub litter in the dry valley and birch litter in the montane forest, which exhibited the highest losses in the pre-freezing period and the freezing period, respectively. In the small mesh size litterbags, the litter mass loss rates of all of the litter types were greatest in the pre-freezing period except for that of cypress litter in the ecotone, which was highest during the thawing period. The litter mass loss rates were generally lowest during the freezing period in all three ecosystems except for in the small mesh size litterbags of birch in the montane forest and cypress in the dry valley, which were lowest during the thawing period, and large mesh size litterbags of fir, which was lowest during the pre-freezing period.

### Contribution of soil fauna to litter-mass loss

**Fig 2:**
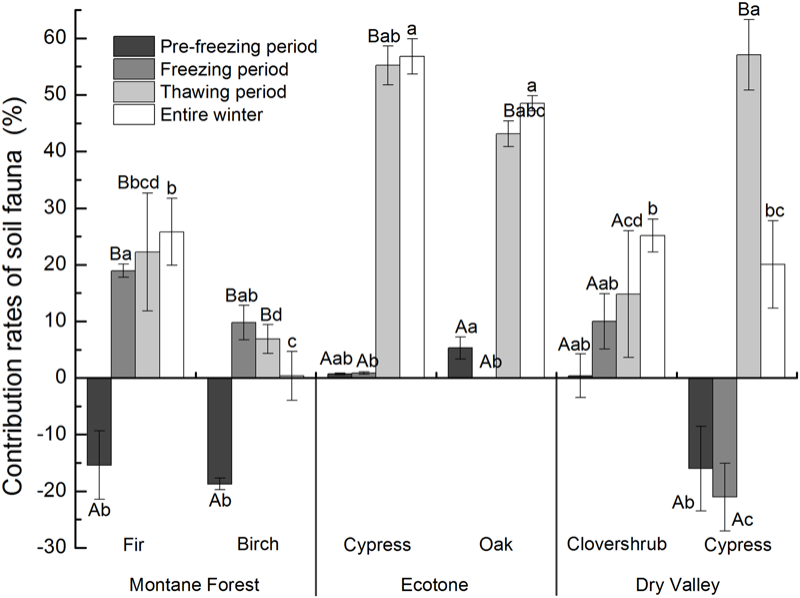
Contribution of soil fauna to the litter-mass loss for different periods and the entire winter (mean ± SE, n = 3). Different capital letters indicate significant differences among different periods for a specific litter type. Different lowercase letters indicate significant differences among litter types for a specific period or the entire winter (*P* < 0.05).

The soil fauna had an obvious influence on the loss of litter mass at all of the sites, but the influence varied significantly among ecosystems. The contribution of soil fauna to litter-mass loss (*P*_*fau*_) in the ecotone, dry valley, and montane forest ranged from 48.5%-56.8%, 20.1%-25.2%, and 0.4%-25.8%, respectively (Fig 2). In all of the ecosystems, the *P*_*fau*_ was highest for the thawing period except for in the birch litter in the montane forest, which had the highest *P*_*fau*_ during the pre-freezing period. For all of the litter types, the lowest values of *P*_*fau*_ occurred during the pre-freezing period except for in the oak litter of the ecotone and birch litter of the montane forest, which had the lowest *P*_*fau*_ during the freezing period and thawing period, respectively. For the dry valley, negative values were obtained for the *P*_*fau*_ of the cypress, fir, and birch litters for the pre-freezing period and cypress litter during the freezing period.

### Temperature/soil moisture and their correlations with the contribution of soil fauna

**Fig 3:**
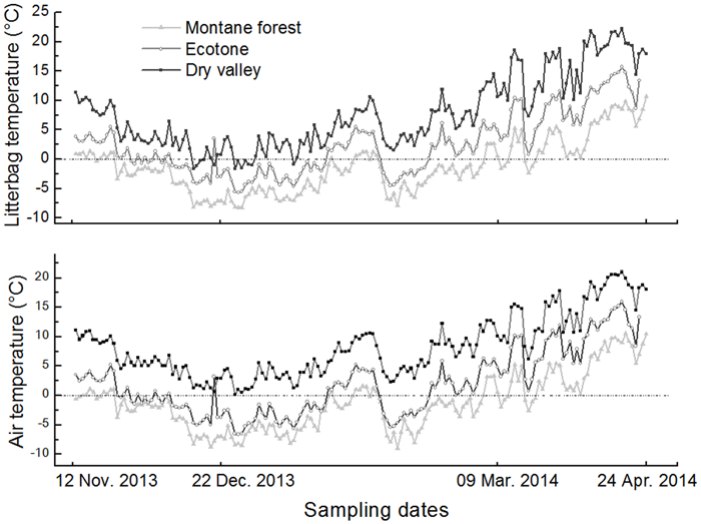
Dynamics of air and litterbag temperatures for the montane forest, ecotone (between the dry valley and montane forest), and dry valley sampling plots from 12 November 2013 to 22 April 2014.

**Fig 4:**
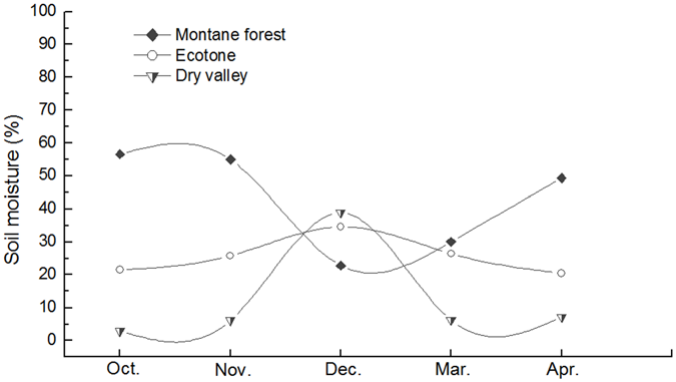
Soil moisture dynamics for the montane forest, ecotone (between the dry valley and montane forest), and dry valley sampling plots from 12 November 2013 to 22 April 2014.

Obvious differences were observed among the temperature of the three sites (Table 2, Fig 3), but the fluctuation patterns of mean temperature were more or less similar for all sites (Fig 3). As to soil moister, however, it showed an adverse pattern between dry valley and montane forest, but a relatively consistent pattern in the ecotone (Fig 4).

**Table 2:** Average temperature (AT), positive accumulated temperature (PAT), and negative accumulated temperature (NAT) for each decomposition stage (°C).

Sampling plots		Pre-freezing period		Freezing period		Thawing period	
		air	litterbags	air	litterbags	air	litterbags
Montane forest	AT	-2.54	-2.44	-3.83	-3.81	4.32	4.03
	PAT	4.83	8.83	7.71	6.04	206.37	192.83
	NAT	-108.86	-108.96	-302.54	-299.21	-7.63	-8.17
Ecotone	AT	-0.01	0.53	-1.00	-0.39	9.46	9.41
	PAT	46.49	54.72	83.20	94.97	435.08	432.85
	NAT	-47.04	-32.92	-160.26	-125.05	-	-
Dry valley	AT	5.93	4.58	5.68	4.55	14.97	16.06
	PAT	243.27	192.04	437.01	356.77	688.66	738.75
	NAT	-	-4.17	-	-6.21	-	-

The *P*_*fau*_ of both the ecotone and dry valley were significantly correlated (*P* < 0.01) with litterbag temperature, whereas the *P*_*fau*_ of the montane forest was not (Table 3). The *P*_*fau*_ of the ecotone and montane forest were significantly (*P* < 0.01) and negatively correlated with soil moisture, whereas the *P*_*fau*_ of the dry valley was positively correlated with soil moisture (Table 3).

**Table 3:** Pearson′s correlation between the loss of mass produced by the soil fauna and litterbag temperature/soil moisture (n = 18).

	Sampling plot	AT	PAT	NAT	Soil moisture
	Montane forest	0.305	0.422	-0.144	-0.571*
*P*_*fau*_	Ecotone	0.979**	0.970**	0.721**	-0.978**
	Dry valley	0.731**	0.709**	0.681**	0.732**

AT: average temperature; PAT: positive accumulated temperature; NAT: negative accumulated temperature.

* *P* < 0.05,

** *P* < 0.01.

## Discussion

The soil fauna had an obvious effect on the litter decomposition in the ecotone during the winter, with the greatest effects observed in the ecotone ecosystem compared with those of the dry valley and montane forest; thus, the results supported our first hypothesis. Additionally, the contributions of soil fauna to the loss of litter mass were highest during the thawing period except for birch litter, which indicated a higher contribution during the pre-freezing period. These results supported our second hypothesis, and indicated that the decomposition processes driven by soil fauna during winter play an important role in providing primary nutrients for plant growth in the subsequent growing season. Moreover, the stronger soil fauna activity in the ecotone provides insight to the more drastic ecological processes that occur in this environment compared with the processes in the adjacent ecosystems.

The process of litter decomposition is primarily controlled by the litter quality [38,39], soil organisms [6], ecosystem vegetation type [40], and environmental conditions [22]. In this study, the litter decomposition throughout the entire winter was the most rapid in the dry valley (regardless of the litterbag mesh size), followed by the ecotone and montane forest (Fig 1). A lack of canopy trees is frequently associated with stronger sunshine and greater exposure to ultraviolet-b radiation (UV-B), and these conditions may contribute to the higher litter-mass loss in the dry valley compared with that of the ecotone and montane forest. In addition, numerous previous studies have documented the photo-degradation of litter [41,42]. What is noteworthy is that litter type may be also an important factor for the significant differences in litter-mass loss rate among differing ecosystems (Table 1, Fig 1), as plant litter identity (litter quality) is always an predominant control on litter decomposition [38]. In the present study, however, litter initial quality showed insignificant influence on the contribution of soil fauna for the entire winter, only several litter quality indexes showed significant (*P* < 0.05) influences on soil fauna contribution rate in certain periods (Table 4). These results suggested that litter quality showed little impact on the contribution rate of soil fauna to litter decomposition, indicating the diverse mass-loss rate and soil fauna contribution rate were mainly attributed to the differences among differing ecosystems.

**Table 4:** Pearson′s correlation between initial litter quality and the contribution of soil fauna to litter decomposition for different periods and the entire winter (n = 18).

Period	TC (g/kg)	TN (g/kg)	TP (g/kg)	C/N	C/P	N/P	Cellulose (%)	Lignin (%)	Lignin/ Cellulose	Lignin/N
Pre-freezing period	-0.032	0.495*	0.203	-0.449	-0.292	0.007	-0.260	-0.136	0.213	-0.355
Freezing period	0.317	0.047	-0.108	0.140	0.203	0.161	-0.500*	0.303	0.232	0.365
Thawing period	-0.338	-0.298	0.243	0.119	-0.462	-0.497*	0.463	-0.497*	-0.275	-0.451
Entire winter	-0.186	-0.020	0.350	-0.031	-0.523*	-0.425	0.015	-0.377	0.001	-0.406

TC: total carbon; TN: total nitrogen; TP: total phosphorus.

*P < 0.05

However, the contribution of soil fauna to litter-mass loss was greater in the ecotone compared with the adjacent dry valley and montane forest ecosystems. This difference may be attributed to the moderate temperature and moisture in the ecotone, which are more favorable for soil faunal activity compared with the conditions of the montane forest and dry valley (Figs 3 and 4). Gonzalez & Seastedt [43] indicated that extreme drought and cold can inhibit the activity of soil fauna; hence, the severe drought conditions in the dry valley and greater degree of freezing in the montane forest may explain the reduced contributions of soil fauna to litter decomposition in these environments, respectively. Furthermore, the correlation analysis indicated that temperature and soil moisture had significant (*P* < 0.01) influences on the contribution of soil fauna to litter-mass loss, and the high degree of variation in decomposition in the ecotone according to specific litter types further confirmed that litter decomposition is strongly correlated with microtopography and vegetation [40,44]. Our results indicate that the contribution of soil fauna to cypress litter-mass loss was greater in the ecotone than in the dry valley, which suggests more active decomposing organisms and related ecological processes in the ecotone.

During litter decomposition, soil fauna can affect the microbial community and physicochemical properties of soil through direct and indirect effects, such as feeding and fragmentation [45], which can further influence the processes governing the cycling of material and flow of energy through the ecosystem. Litterfall often occurs in early winter before the soil is completely frozen [46]. Although fresh litter is rich in easily decomposable components [47], the effects of the soil fauna on litter decomposition may have shown a delay because it was difficult to collect the litter in the litterbags. In addition, the decrease in temperature during the pre-freezing period can lead to the death of a certain portion of the soil faunal community, which further reduces their contribution to litter decomposition [30]. However, the contribution of soil fauna to the birch litter-mass loss was highest during the pre-freezing period, which was generally associated with the inhibition of soil fauna. This inhibition may have been caused by the addition of allochthonous litter and/or by reductions in the microbial activity caused by soil fauna [45]. Similar effects were also observed in litterbags with fir and cypress litter in the montane forest and dry valley, respectively (Fig 1).

The results of our study suggest that the contribution of soil fauna to litter-mass loss was higher during the freezing period than the pre-freezing period. Continuous decreases in temperature completely freeze the soil during the freezing period [15,22]. Nevertheless, soil faunal activities do not cease [48]. Numerous soluble components are lost during the pre-freezing period, and freezing events can significantly alter litter quantity and quality, thus accelerating the decomposition of the remaining recalcitrant components [49]. In addition, soil fauna can play an important role during litter decomposition during freezing periods because snow cover can provide a relatively stable environment for soil faunal activities [50,51]. However, in the dry valley, the effect of soil fauna on the cypress litter was inhibited by the freezing period. This inhibition may have been caused by the freezing of foliar litter during the freezing period, which limited the ability of the soil fauna to feed [52,53]. Moreover, a recent study has suggested that soil fauna can feed on soil microorganisms during freezing periods to compensate for the lack of food, which might decrease litter decomposition during this period [45].

With increases in temperature during the thawing period, the activity of soil fauna and microbes increase. The results of our study also indicate that the relative contribution of soil fauna to litter-mass losses were highest during the thawing period. Furthermore, after the decomposition of recalcitrant components during the freezing period, a greater abundance of small molecular substances are available for soil organisms [3], which may promote the effects of soil fauna on litter decomposition. However, the results indicate that the contribution of soil fauna to birch litter-mass losses was lowest during the thawing period. This lower contribution may be related to the initial birch litter properties because plant species is an important factor that explains variations in the decomposition rates among litter types [38,39].

Moreover, although using fine mesh size on the bottom of large litterbag may reduce the activities of certain members of soil fauna during litter decomposition, such a method may be more acceptable as a coarse mesh size on the soil side can result in even bigger errors when calculating litter mass loss. Furthermore, as some very small soil fauna such as nematodes can enter the 0.04 mm mesh size litter bags [20], the present study may have ignored the effect of such microfauna, which need further investigations in further studies.

In summary, obvious contributions of soil fauna were observed in winter during litter decomposition in the ecotone, dry valley, and montane forest. Compared with the adjacent ecosystems, ecotone showed higher soil fauna contributions during litter decomposition in winter, which provide efficient evidences in understanding the more intense flux of material and energy. However, absolute litter mass loss was higher in dry valley than that in ecotone and montane forest, and higher soil fauna contributions were observed in thawing period with better temperature environment. The observations here suggest that temperature may be the primary factor controlling foliar litter decomposition, although more active soil fauna in the ecotone. Even so, the expanding dry valley and winter warming might promote litter decomposition, but limit the activity of soil fauna in this region.

## Supporting Information

S1 Dataset Dataset used in the present study. (XLSX)
